# Effectiveness of enfuvirtide in a cohort of highly antiretroviral-experienced HIV-1-infected patients in Mexico

**DOI:** 10.1186/s12981-014-0040-9

**Published:** 2014-12-31

**Authors:** Gloria Huerta-García, Marcelino Chavez-García, José Antonio Mata-Marín, Jorge Sandoval-Ramírez, Juan Domínguez-Hermosillo, Ana Lourdes Rincón-Rodríguez, Jesús Gaytán-Martínez

**Affiliations:** Departamento de Infectología Pediátrica, Hospital de Pediatría, Centro Médico Nacional “Siglo XXI”, IMSS, México City (Distrito Federal), México; Unidad Médica de Alta Especialidad No. 25, IMSS, Monterrey, Nuevo León México; Departamento de Infectología adultos, Hospital de Infectología, Centro Médico Nacional “La Raza”, IMSS, México City (Distrito Federal), México; Servicio de Infectología, Hospital de Pediatría Centro Médico Nacional Siglo XXI, Avenida Cuauhtémoc 330, Cuauhtémoc, Doctores, Ciudad de México, DF 06720 México

**Keywords:** Drug tolerability, Enfuvirtide, HIV, Treatment- experienced, Virologic suppression

## Abstract

**Background:**

Treatments in patients with multidrug resistance often involve the use of multiple agents with partial antiviral activity and overlapping metabolic toxicities. Enfuvirtide is therefore a welcome addition to the antiretroviral management of patients with multiclass resistant virus, given the low risk of systemic toxicities and novel mechanism of action relative to existing drug classes.

The aim of this study was to evaluate the effectiveness of ENF plus optimized background regimen (OBR) in a Mexican cohort of highly HIV-1 ARV-experienced patients.

**Methods:**

Prospective cohort of treatment-experienced HIV-1-infected adults with virological failure who started therapy with an ENF-containing regimen. The effectiveness of ENF treatment was evaluated with percentages of undetectable HIV-1 RNA viral load after 24 and 48 weeks of treatment, and changes in CD4+ cell counts.

**Results:**

Forty patients >18 years were included. After 24 weeks of treatment, 91% of patients had HIV-1 RNA viral load <400 copies/mL and 65.8% had <50 copies/mL. At week 48 of treatment, 81.4% of the patients had HIV-1 RNA <400 copies/mL and 55.5% had <50 copies/mL; in both cases *p* <0.0001 compared to baseline. Increase CD4+ cells were also statistically significant at weeks 24 and 48 with respect to the baseline. Pain at the site of injection was the main adverse event in 100% of patients.

**Conclusion:**

Our study provides clinically important evidence of the effectiveness and safety of ENF in highly ARV-experienced HIV-1-infected patients. These findings strengthen the results of previous randomized controlled trials with this agent.

## Background

Although rates of multiple regimen failure have decreased dramatically over the past decade, mortality rates for those who have experienced failure of at least two regimens remain high [1]. Some studies have shown that patients with multidrug failure or multidrug resistance have an overall poor prognosis compared with those who experience more limited drug failure [2,3]. Enfuvirtide (ENF, also known as T-20) is a novel, synthetic, 36-amino-acid peptide that binds to a region of the envelope glycoprotein 41 of human immunodeficiency virus (HIV) type 1 (HIV-1), which is involved in the fusion of the virus with the membrane of the CD4+ host cell. This agent exhibits potent and selective inhibition of HIV-1 in vitro without cytotoxicity [4].

ENF was approved in 2003 as a salvage therapy agent following the success of the Phase III TORO (T-20 vs Optimized Regimen Only) clinical trials. TORO 1 (United States, Canada, Mexico, and Brazil) and TORO 2 (Australia and Europe) assessed the safety and efficacy of ENF plus an optimized background (ENF + OB) versus optimized background alone (OB) in approximately 1000 treatment-experienced patients (n = 663 ENF + OB and n = 334 OB) [5,6]. RESIST 1 and RESIST 2 trials involved 1509 patients with treatment failure with at least two protease-inhibitors [7]. Patients were randomized to take OB plus ritonavir-boosted tipranavir or OB plus a ritonavir-boosted comparator protease inhibitor (CPI). Tipranavir/ritonavir had greater response than the control arm.

When patients had ENF as a part of their OB, response was improved in both groups. At 48 weeks, 58.5% of enfuvirtide-naïve patients in the tipranavir/ritonavir arm who received enfuvirtide had a treatment response compared with 25.6% of enfuvirtide-naïve patients randomized to the control arm and receiving enfuvirtide.

Clinical trials POWER 1 and POWER 2 randomized highly treatment-experienced patients to ritonavir-boosted darunavir plus OB or CPI plus OB [8]. At 48 weeks, 61% of patients in the darunavir/ritonavir arm had a reduction of plasma viral load of around 1 log_(10)_ copies/mL versus 15% in the CPI arm. This result was more pronounced in enfuvirtide-naïve patients who received enfuvirtide plus boosted darunavir and OB since 81% had a decline in plasma viral load of >1 log_(10)_ copies/mL, versus 56% of patients taking boosted darunavir and OB without enfuvirtide. Few real life trials have demonstrated the effectiveness of ENF in highly HIV-1 antiretroviral (ARV)-experienced patients. The aim of this study was to evaluate the effectiveness of ENF plus OB regimen (OBR) in a Mexican cohort of highly HIV-1 ARV-experienced patients.

## Methods

### Design

We developed a prospective cohort of treatment-experienced HIV-1-infected adults who started therapy with an ENF-containing regimen. The first end point occurred after patients completed 24 weeks of treatment. Secondary end point was the evaluation to 48 weeks of treatment.

### Patients

Patients were >18 years of age with confirmed HIV-1 infection by Enzyme-Linked ImmunoSorbent Assay *(*ELISA) and Western blot (WB), virological failure, mutations detected from three classes of antiviral drugs and naïve to ENF. Patients had previous treatment with at least three classes of antiretroviral (ARV) drugs including Nucleoside analog reverse-transcriptase inhibitors (NRTIs), Non-nucleoside reverse-transcriptase inhibitors (NNRTIs), and Protease inhibitors (PIs), only one patient had history of integrase inhibitor use (IIN), with mutation resistance documented for each class.

An individualized OBR was chosen for each patient. The regimen included three to five ARV agents, with ≥2 fully active drugs according to HIV-1 resistance testing and previous antiretroviral drug experience.

### Measurements

Clinical history regarding ARV regimens, CD4+ cells count, HIV-1 RNA viral load, and serum laboratory parameters at the beginning of the therapy with ENF (baseline) and at 24 and 48 weeks later was recorded.

Mutations were assessed from plasma HIV-1 *pol* sequences using the Stanford database. The presence of resistance was defined according to Stanford HIVdb (SS) ranges as follows: 0–9 = susceptible; 10–14 = potential low-level resistance; 15–29 = low-level resistance; 30–59 = intermediate resistance; and ≥60 = high-level resistance.

The genotype sensitivity score (GSS) was defined as the total number of drugs (excluding enfuvirtide) in a participant’s optimized background antiretroviral regimen to which their HIV isolate had genotypic sensitivity, as deduced from gene sequence and mutation analyses. This was calculated based on the drug resistance scores extracted from the Stanford HIV data base (HIVdb). Each antiretroviral drug (ARV) was assigned a score according to the five-level Stanford HIVdb interpretation: 1.00, 0.75, 0.50, 0.25 and 0.00 for susceptible, potential low-level resistance, low-level resistance, intermediate resistance and high-level resistance. The GSS was the sum of all scores for all ARVs in the patient’s OBR regimen. The arithmetic sum of the individual score for the specific drugs provided the total GSS of that treatment. We classified the total GSS score in the following categories: 0 to 1, 1 to 2, or ≥2. The 0 to ,1 group contains viral sequences almost entirely resistant to the drugs in their regimen, and the ≥2 group contains viral sequences susceptible to ≥2 drugs given in their OBR regimen [9].

The effectiveness of ENF treatment was evaluated with percentages of undetectable HIV-1 RNA viral load after 24 and 48 weeks of treatment. We also evaluated changes in CD4+ cell counts.

Tolerability endpoints were drug tolerability, death, and immune reconstitution and inflammatory syndrome reactions (ISRs). Other evaluations were changes in fasting lipids levels (total cholesterol, triglycerides), and creatinine from baseline to weeks 24 and 48.

### Statistical analysis

Baseline characteristics were summarized using medians and interquartile ranges (IQR) for continuous variables and proportions for categorical variables. Nonparametric paired tests were used to evaluated CD4 counts and viral load changes. Descriptive statistics were used to evaluate changes in CD4+ cell count and HIV-RNA viral load from baseline. The following statistics were calculated for continuous variables: mean, standard deviation, median with IQR. For categorical variables, number of values in each category and percentage of the values with regard to number of patients in the study population were calculated. Explorative statistical methods were used regarding the efficacy endpoints and changes in safety-relevant laboratory parameters. Significance changes from baseline were tested using the Wilcoxon signed-rank test. All analyses were carried out using SPSS software (version 17; SPSS Inc, Chicago, IL).

## Results

We included 40 patients; 37 patients fulfilled the inclusion criteria for the 24-week analyses and 27 of those patients were followed through the 48-week analyses. Two patients died because of advanced disease at the beginning of the ENF regimen (<12 weeks). These deaths were not associated with ENF treatment.

The median age of the overall cohort at enfuvirtide initiation was 44.8 years (SD ±8.79) and 89.5% were men. CDC Class C AIDS was found in 64.8% of patients and median of previous ARV treatment was 5 (IQR 5–7) (Table 1). When we analyze OBR according to the Stanford HIVdb, we did not find difference between the SS of OBR with virological failure and the one who got <50 copies/mL

**Table 1:** **Baseline characteristics and optimized background**

	**DRV/rtv + ENF+ OBR (n = 21)**	**TPV/rtv + ENF + OBR (n = 12)**	**Other + ENF + OBR (n = 4)**	**All patients (N = 37)**
**Demographics**				
**Male**	19	10	4	33
**Mean age, years (±SD)**	43 (7.7)	47 (11.2)	45 (6.2)	44.8 (8.79)
**Disease characteristics**				
**Median duration of infection (range)**	13 (8–22)	13 (5–19)	14 (5–19)	13 (5–22)
**Median baseline log**_**10**_ **HIV RNA (range)**	4.22 (1.80-5.76)	4.18 (3.45-5.59)	4.20 (2.54-5.00)	4.22 (1.80-5.76)
**Median CD4+ cells count, cell per mL (range)**	177 (20–508)	301 (74–561)	119 (8–339)	224 (8–561)
**CDC class, n (%)**				
**A**	3 (14.2%)	1 (8.3%)	0	4 (10.8%)
**B**	6 (28.5%)	2 (9.5%)	1 (25%)	9 (24.3%)
**C**	12 (57.1%)	9 (42.8%)	3 (75%)	24 (64.8%)
**History of antiretroviral treatment**				
**Median of previous ARV treatments (range)**	5 (3–10)	5.5 (4–12)	5.5 (5–7)	5 (3–12)
**Baseline resistance**				
**Primary IAS-USA** **[** 10 **]** **PI mutations (range)**	2 (0–5)	2 (0–6)	3 (0–5)	2 (0–6)
**PI RAMs**	3 (1–7)	4.5 (2–7)	4 (1–7)	4 (1–7)
**NRTI RAMs**	5 (0–8)	6 (4–7)	4 (2–4)	5 (0–8)
**NNRTI RAMs**	1 (0–3)	1 (0–4)	2 (0–2)	1 (0–4)
**Number of active ARV in OBR, n (%)**				
0	0	1 (8.3%)	0	1 (2.7%)
1	4 (19%)	8 (66.6%)	1 (25%)	13 (35.1%)
≥2	17 (81%)	3 (25%)	3 (75%)	23 (62.2%)
*GSS OBR, Median (IQR)	2.0 (1.50-2.25)	1.13 (1.0-1,38)	2.0 (1.63-2.13)	1.50 (1.25-2.00)
**GSS OBR, n (%)**				
**≥2**	12 (57.1%)	0 (0%)	3 (75%)	15 (40.5%)
**1 to <2**	9 (42.8%)	11 (91.6%)	1 (25%)	21 (56.8%)
**0 to <1**	0 (0%)	1 (8.33%)	0	1 (2.7%)
**Three most used ARV in OBR, n (%)**				
TDF	7 (33.3%)	7 (58%)	3 (75%)	17 (46%)
RAL	19 (90%)	9 (75%)	4 (100%)	32 (86%)
ETV	4 (19%)	0	1 (25%)	5 (13.5%)

DRV = Darunavir, rtv = Ritonavir, TPV = Tipranavir, RAL = Raltegravir, TDF = Tenofovir, ETV = Etravirine, PI = proteasa inhibitor, GSS = genotypic susceptibility score, *Genotypic score according to Stanford HIV db.

.

The median calculated GSS of the current regimen without taking into account enfuvirtide was 1.5 (IQR 1.24-2.0), 1.5 (IQR 1.0-1.63) in patients with failure and 2.0 (IQR 1,25-2,25) in patients with out failure, P = 0.07. Fifteen (40.5%) patients had a GSS ≥2. Per group, DRV/r and non-IP OBR group had the mayor proportion of the patients with GSS ≥2; TPV/r had majority of GSS 1 to <2 regimens (Table 1). None of the ORB groups has a relationship between the lower GSS and major possibility of failure (P = 0.23 for DRV/r OBR; P = 0.56 for TPV/r OBR, and insufficient data for NonIP OBR group). The GSS 1- ≤ 2 at week 48 was associated with viral load grater than 50 copies/ml (p = 0.03), but not with grater than 200 copies/mL (p = 0.12).

At baseline, the median HIV-1-RNA viral load was 17,677 copies/mL [4.24 log_(10)_] (IQR 3,702–92,555 copies/mL [3.5–4.9 log_(10)_]). After 24 weeks of treatment, 91% of patients (n =37) had HIV-1 RNA viral load <400 copies/mL and 65.8% had <50 copies/mL; median, <50 copies/mL [<1.6 log_(10)_] (IQR <50–137 copies/mL [<1.6–2.1 log_(10)_]). At week 48 of treatment, 81.4% of the patients (n = 27) had HIV-1 RNA <400 copies/mL and 55.5% had <50 copies/mL; median, <50 copies/mL [<1.6 log_(10)_] (IQR <50–185 copies/mL [<1.6–2.26 log_(10)_]), in both cases (*p* <0.0001 compared to baseline).

At baseline, the median and interquartile range for the CD4+ cells count were 225 cells/μL (110–478 cells/μL), whereas at weeks 24 and 48 the values were 301 cells/μL (216–549 cells/μL) and 443 cells/μL (260–585 cells/μL), respectively. These increases were both statistically significant (*p* <0.0001).

For fasting lipid profiles (Total Cholesterol [TC], and Triglycerides [TG]), TG showed a significant increase (*p* = 0.016) from baseline 204 mg/dL (159–290 mg/dL) to 258 mg/dL (220–374 mg/dL) at week 24, but there was no significant increase at 48 weeks [267 mg/dL (166–302 mg/dL)] (*p* = 0.372). TC showed no significant increase at either period. There was no significant increase in creatinine compared to baseline values (Table 2).

**Table 2:** **End points after 24 and 48 weeks of treatment**

**Outcomes**	**Median (IQR)**			***p value***
	**Baseline**	**Week 24**	**Week 48**	**24/48 weeks**
**CD4+ cells count**	225 (110–478)	301 (216–549)	443 (260–585)	<0.0001*
**HIV-1 RNA viral load**	17,677 (3,702–92,555)	50 (<50–137)	50 (<50–185)	<0.0001*
**Cholesterol (mg/dL)**	174 (154–196)	193 (156–230)	195 (169–217)	0.158/0.170
**Triglycerides (mg/dL)**	204 (159–290)	258 (220–374)	267 (166–302)	0.016/0.372
**Creatinine (mg/dL)**	0.9 (0.77–1.0)	1.0 (0.9–1.1)	1.0 (0.8–1.1)	0.157/0.091

*At both 24 and 48 weeks.

Pain at the site of injection was the main adverse event in 100% of patients. The other adverse event was the presence of subcutaneous nodules at the injection site in 45.4% of the patients. Whereas 19% of patients developed ISRs, they did not required therapy discontinuation.

## Discussion

ENF represented an important breakthrough. It was the first approved ARV agent belonging to a new drug class and hence cross-resistance with previous agents was not a problem. ENF is not used usually in HIV-naïve patients except among highly ARV-experienced patients who do not have another therapy option.

Our study provides additional published data describing the effectiveness and safety of ENF among ARV-experienced patients in a clinical setting. Therapy with ENF-based regimens was associated with high levels of HIV-1 RNA viral load suppression through 6 and 12 months follow-up, with 91% and 81% in highly treatment-experienced patients with viral load <400 copies/mL, and 65.8% and 55.5% <50 copies/mL, respectively, and was seems to be independent of genotypic sensitivity score according HIV Stanford database of OBR.

In addition, therapy with ENF was associated with a median increase in CD4+ cell counts after 6 months and even greater at 12 months, which is clinically relevant given the patients’ extensive treatment history and limited options for background therapy.

Because of the disparity of patient regimens, it is difficult to determine the contribution made by an individual agent within the regimen, although, in the mutations analysis, we can observe that PI score was highly compromised; then the role of ENF in reducing viral load in these patients was likely substantial given their large degree of baseline resistance to the alternative classes.

As predictors of therapeutic success we use GSS, predictor derived from viral genotype. Including genotype information into treatment outcome prediction is challenging because of the large number of observed mutations and the complexity of the genotype-phenotype relationship, in our study GSS value was not associated with failure but sample size is not enough for this analysis, and much less for the analysis by OBR groups.

In addition, the clinical efficacy of ENF has been demonstrated in several studies, in which it was shown to be superior with an optimized ARV background versus optimized background alone (T-20 vs. Optimized Regimen Only [TORO] studies) in highly treatment-experienced patients [5,6]. Outside of clinical trials, there are few published studies with similar results of effectiveness, durability, and safety of ENF among ARV-experienced patients in clinical settings. Loutfy *et al*. found that therapy with ENF-based regimens for a minimum of 2 months was associated with a 2.53 log_(10)_ viral load decrease through 12-month follow-up in a retrospective cohort of 67 treatment-experienced patients [11]. Belperio *et al*. described a cohort of treatment-experienced, older, HIV-infected patients who received ENF-based regimens: 41% and 55% achieved viral load <400 copies/mL at 6 months and 2 years, respectively (analysis of unique time period of ENF use before the availability of newer oral agents indicated for treatment experienced patients) [12]. In a retrospective monocentric cohort of 18 patients who started ENF and completed at least 3 months of therapy, 11 (61%) patients had HIV-1 RNA viral load <400 copies/mL, among whom eight (44%) patients had <50 copies/mL in the first 3 months. The median increase in CD4+ cell count was 159 cells/mm^3^ (range, 25 to 301) and the median decrease in HIV-1 RNA viral load was 2.5 ± 1.4 log_(10)_ after 12 months of ENF-based regimen [13].

As to regimen tolerability, a mild increase in plasma lipid levels during the initial 24 weeks of treatment was not sustained at the final follow-up, at variance with reports elsewhere. Elevation of lipids at baseline may be attributed to those previous and currently exposure to PI-based regimens [7,14].

For the injection site reaction, observed at 6 and 12 months, our findings are consistent with previous studies [15], although the percentages reported by Cohen *et al*. were higher [16].

ENF was fairly well tolerated by patients in this study. Although 19% of patients developed ISRs, it was not associated with discontinuation therapy. These data differ from those observed in the TORO studies, in which ISRs were observed in 98% of patients receiving ENF, leading to treatment discontinuation in 4.4% of the study participants [5,6] and Loutfy *et al*. who reported 52% of their patients developed ISRs, and 14.1% of the entire cohort discontinued therapy as a result of ISRs [11].

Despite being a retrospective, descriptive design study with a small number of patients and lack of a control group or randomization, this Mexican study also demonstrates that ENF is a valuable therapy option in highly ARV-experienced HIV-1-infected patients who have advanced disease, resistance to traditional ARV drug classes, and clinical progression with very few or no treatment options.

Currently, there is no new different drugs families in HIV, regarding drugs with different mechanism of action and no cross-resistance mutations, some people with HIV multi-drug resistance has only integrase inhibitors and sometimes CCR5 antagonist or partial activity of a PI to integrate an OBR; then, ENF will continue as part of salvage therapy in highly ARV-experienced patients.

ENF has been successfully used to produce durable reductions in viremia even in patients with multi-drug resistance. The likelihood of a sustainable virologic and immunologic responses is maximized when the patient has at least 2 other active agents besides ENF in the OBR, CD4+ cells counts are greater than 100 cells/mm^3^, and RNA HIV-1 plasma viral load is less than 100,000 copies/ml.

Despite its adverse injection site effects, the addition of ENF to an optimized background antiretroviral regimen did not exacerbate toxicities associated with antiretroviral therapy, and patients treated with enfuvirtide experienced a significantly lower incidence of diarrhea and other gastrointestinal side effects.

In conclusion, our study provides clinically important evidence of the effectiveness and safety of ENF in highly ARV-experienced HIV-1-infected patients. These findings strengthen the results of previous randomized controlled trials with this agent.
